# Retinopathy, Neuropathy, and Subsequent Cardiovascular Events in Patients with Type 2 Diabetes and Acute Coronary Syndrome in the ELIXA: The Importance of Disease Duration

**DOI:** 10.1155/2018/1631263

**Published:** 2018-12-16

**Authors:** Jelena P. Seferovic, Rhonda Bentley-Lewis, Brian Claggett, Rafael Diaz, Hertzel C. Gerstein, Lars V. Køber, Francesca C. Lawson, Eldrin F. Lewis, Aldo P. Maggioni, John J. V. McMurray, Jeffrey L. Probstfield, Matthew C. Riddle, Scott D. Solomon, Jean-Claude Tardif, Marc A. Pfeffer

**Affiliations:** ^1^Cardiovascular Division, Brigham and Women's Hospital, Harvard Medical School, Boston, MA, USA; ^2^Massachusetts General Hospital, Harvard Medical School, Boston, MA, USA; ^3^Estudios Clínicos Latinoamérica, Rosario, Argentina; ^4^McMaster University, Hamilton, ON, Canada; ^5^Rigshospitalet Copenhagen University Hospital, Copenhagen, Denmark; ^6^Sanofi U.S., Bridgewater, NJ, USA; ^7^Research Center of the Italian Association of Hospital Cardiologists, Florence, Italy; ^8^British Heart Foundation Cardiovascular Research Centre, University of Glasgow, Glasgow, UK; ^9^University of Washington Medical Center, Seattle, WA, USA; ^10^Oregon Health and Science University, Portland, OR, USA; ^11^Montreal Heart Institute, Université de Montréal, Montreal, Canada

## Abstract

**Introduction:**

We investigated the association of diabetic retinopathy and neuropathy with increased risk of recurrent cardiovascular (CV) events in 6068 patients with type 2 diabetes mellitus (T2DM) and recent acute coronary syndrome (ACS) enrolled in the Evaluation of Lixisenatide in Acute Coronary Syndrome (ELIXA).

**Methods:**

History of retinopathy and neuropathy as well as duration of T2DM were self-reported at screening. Proportional hazards regression models were used to assess relationships between retinopathy, neuropathy, and recurrent CV events.

**Results:**

At screening, retinopathy and neuropathy were reported in 10.7% and 17.5% of patients, respectively, while 5.7% reported both. When adjusted for randomized treatment only, both retinopathy and neuropathy were associated with a primary composite outcome (CV death, nonfatal MI, stroke, or hospitalization for unstable angina) (retinopathy: HR 1.44, 95% CI 1.19–1.75; neuropathy: HR 1.33, 95% CI 1.12–1.57), CV composite (CV death, nonfatal MI, stroke, hospitalization for heart failure (HF)) (retinopathy: HR 1.57, 95% CI 1.31–1.88; neuropathy: HR 1.38, 95% CI 1.19–1.62), myocardial infarction (retinopathy: HR 1.38, 95% CI 1.08–1.76; neuropathy: HR 1.26, 95% CI 1.02–1.54), HF hospitalization (retinopathy: HR 2.03, 95% CI 1.48–2.78; neuropathy: HR 1.71, 95% CI 1.30–2.27), and all-cause mortality (retinopathy: HR 1.65, 95% CI 1.28–2.12; neuropathy: HR 1.43, 95% CI 1.14–1.78). When included in the same model, and adjusted for T2DM duration, there were no independent associations of either with CV outcomes, while T2DM duration remained strongly associated with all outcomes. Addition of demographic characteristics and CV risk factors did not further alter these relationships.

**Conclusions:**

In patients with T2DM and recent ACS, a history of retinopathy and/or neuropathy and longer T2DM duration could be considered clinical markers for high risk of recurrent CV events. This trial is registered with the ELIXA (Evaluation of Lixisenatide in Acute Coronary Syndrome), ClinicalTrials.gov registration number NCT01147250.

## 1. Introduction

Cardiovascular (CV) risk in type 2 diabetes mellitus (T2DM) was associated with disease duration and severity of hyperglycemia in many studies [1–3]. In the Framingham Heart Study, duration of T2DM significantly and positively related to the risk of coronary heart disease mortality, but not morbidity, or CV disease (CVD) morbidity or mortality [2]. The Action in Diabetes and Vascular Disease: Preterax and Diamicron Modified Release Controlled Evaluation (ADVANCE) trial, on the contrary, showed that T2DM duration was independently associated with the risk of macrovascular complications and death [3]. In a meta-analysis of five trials, intensive glycemic control led to a reduction of nonfatal myocardial infarction (MI) incidence by one-sixth, with no significant effect on the incidence of nonfatal stroke, and both CV or all-cause mortality [4].

Additionally, duration of T2DM and severity of hyperglycemia are strong and consistent risk factors for the development and progression of microvascular diabetic complications—retinopathy (DR), neuropathy (DN), and nephropathy [5–8]. For example, in the very large and diverse T2DM population in ADVANCE, T2DM duration was independently associated with the risk of DR and nephropathy [3]. In the Maastricht Study, a T2DM-enriched population-based cohort study prediabetes, T2DM, and measures of hyperglycemia were independently associated with DR and DN [9]. Improved metabolic control was associated with 25% risk reduction of microvascular composite endpoint in the United Kingdom Prospective Diabetes Study (UKPDS) [10]. Similar effect has been shown for individual complications, DR [5, 11], DN [6, 11], and nephropathy [7, 11, 12].

The association of diabetic nephropathy and increased risk of CVD has been revealed in multiple patient cohorts. Data from United States community-based study demonstrated that individuals with diabetic nephropathy were at four- and threefold higher risk for CV death and all-cause death, respectively, compared to those without [13]. In the ADVANCE study, both albuminuria and reduced estimated glomerular filtration rate (eGFR) were independently and continuously associated with the risk for CV and kidney outcomes in T2DM patients [14]. Similarly, among patients with type 1 diabetes followed for over 30 years, the development of diabetic nephropathy was associated with higher risks of CVD and renal events. However, this effect was almost entirely eliminated by adjustment for updated mean HbA1c which led to the conclusion that glycemic exposure correlates very strongly with CVD and mortality, and that this is partly mediated by hyperglycemia-induced renal disease [15]. Steno-2 study, including T2DM patients with microalbuminuria, showed that long-term intensified therapy reduced the risk of CV events, as well as diabetic nephropathy, DR, and DN [16]. Also, the risk of microalbuminuria has been shown to increase with T2DM duration [17].

Potential associations, of DR and DN with CV outcomes, whether causal or only predictive, have received less attention. We therefore investigated the association of DR and DN with increased risk of recurrent CV events, in patients with a recent acute coronary syndrome (ACS).

## 2. Materials and Methods

### 2.1. Study Design and Patients

The ELIXA (Evaluation of Lixisenatide in Acute Coronary Syndrome) was a randomized, double-blind, placebo-controlled trial designed to assess the effects of lixisenatide added to current T2DM therapy on CV morbidity and mortality in 6068 patients with a recent ACS.

We examined the primary composite (CV death, nonfatal MI, stroke, or hospitalization for unstable angina), CV composite (CV death, nonfatal MI, stroke, and hospitalization for heart failure (HF)), each of its components, and all-cause mortality. Details of the trial design, entry criteria, and the main results have been reported previously [18, 19].

For this post hoc analysis, all 6068 ELIXA participants were included. Self-reported historical data on DR and DN were collected at screening. Patients were asked to answer “yes,” “no,” or “unknown” on the presence of DR and/or DN. If DR was present, date of diagnosis was recorded, as well as information on photocoagulation and vitrectomy. However, these interventions were not analyzed further due to a small number of events. Presence of DN was defined as a report of either sensory/motor or autonomic neuropathy. Only “yes” responses were used to define the exposure variables for all subsequent analyses. Blood samples included in this analysis were done at screening by a central laboratory. Duration of T2DM was evaluated based on medical record review or self-report at the screening visit.

### 2.2. Statistical Analysis

Baseline characteristics of patients were stratified by the presence of DR and/or DN. Descriptive data are presented as the mean ± standard deviation for normally distributed variables and as median (25–75th percentile) for nonnormally distributed variables. Categorical variables are expressed as proportions and were compared by the chi-square test, while continuous variables were compared using *t*-tests or Wilcoxon rank-sum tests, as appropriate. Two proportional hazards regression models were used to assess the association between DR, DN, and recurrent CV events. Multivariable proportional hazards models were used to assess the association between DR, DN, and primary composite endpoint, CV composite endpoint, components, and all-cause mortality. Both DR and DN were included in the first model and adjusted for the duration of T2DM. In the second model, demographic characteristics and CV risk factors (age, sex, race, body mass index (BMI), baseline HbA1c, smoking, history of hypertension (HT), heart rate, total cholesterol, low-density lipoprotein (LDL) cholesterol, and triglycerides) were added to the previous model. Both models were adjusted for randomized study treatment. Predictors of DR and DN were determined from multivariable logistic regression model using forward stepwise selection including all variables in Table 1. Two-sided *p* values < 0.05 were considered significant. No adjustment was made for multiple comparisons. Analyses were performed using the Stata version 13.1 (StataCorp., College Station, TX, USA).

## 3. Results

### 3.1. Baseline Characteristics

**Figure 1:**
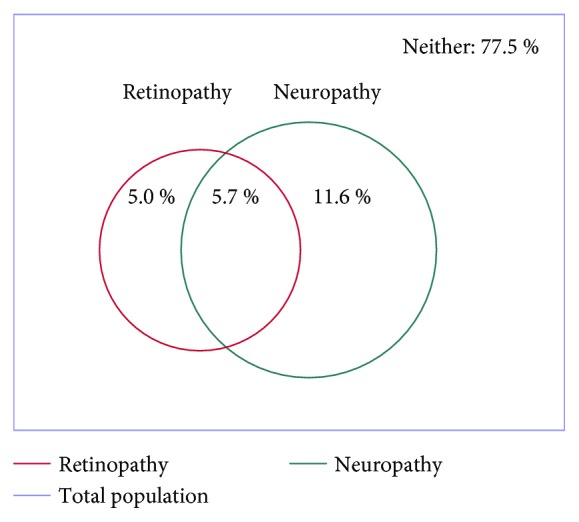
Prevalence of retinopathy and/or neuropathy.

Demographic and clinical characteristics of the 6068 patients are shown in Table 1. In the whole population, the mean age was 60.3 years and known T2DM duration was 7.4 years. However, the reported duration of T2DM varied widely, with 15.9% of participants having known T2DM for less than 1 year, 21.3% ≤5 years, 22.4% >1–≤5 years, and 40.4% longer than10 years (Supplementary Figure 1). Of the whole population, 1363 (22.5%) reported DR and/or DN. DR was reported in 651 (10.7%) patients, DN in 1060 (17.5%) patients, and both in 348 (5.7%) patients (Figure 1). Of 651 who reported DR, 159 patients (24.4%) had prior photocoagulation, and 32 patients (4.9%) had vitrectomy.

**Figure 2:**
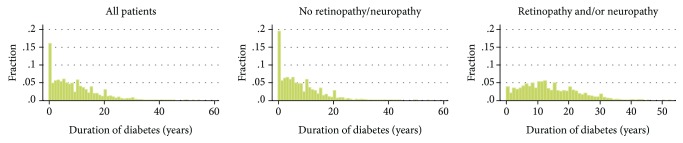
Distribution of T2DM duration in all patients, patients with no retinopathy/neuropathy and patients with retinopathy and/or neuropathy.

Patients who had DR and/or DN were significantly older (mean 62.3 vs. 59.7 years) and had longer known duration of T2DM (mean 12.4 vs. 6.0 years) than those with neither complication. Smaller proportions of patients with DR and/or DN reported shorter T2DM duration of <1 year (3.7% vs 19.4%) and ≤5 years (12.0% vs. 24.0%). The distribution of T2DM duration in all patients, as well as in those with and without retinopathy and/or neuropathy, is shown in Figure 2. Participants with DR and/or DN also had higher BMI, were also more likely to be nonsmokers, and had significantly higher glycated hemoglobin, fasting plasma glucose, and total and LDL cholesterol. The subgroup with DR and/or DN also had more evidence of renal disease reflected as increased albumin-to-creatinine ratio in comparison with those without DR and/or DN. Also, they were more likely to have history of CV disease (HT, HF, stroke, peripheral arterial disease, and atrial fibrillation). Finally, patients with DR and/or DN used more frequently metformin and insulin, while other glucose-lowering agents were similarly distributed among groups (Table 1).

### 3.2. Retinopathy and/or Neuropathy and CV Outcomes

In univariate analysis, DR was significantly associated with primary and CV composite endpoint, all-cause and CV death, and all CV events except stroke (*p* = 0.068), while DN was associated with primary and CV composite endpoint, all-cause death, and all nonfatal CV events, but not CV death (*p* = 0.08, Table 2). When both DR and DN were included in the same model, along with T2DM duration, there were no independent associations of either with any of the outcomes, while duration of T2DM remained highly significantly associated with all outcomes (Table 2). Furthermore, the addition of demographic characteristics and CV risk factors to the previous model resulted in neither DR nor DN being associated to any of the outcomes, but identified duration of T2DM as an independent predictor of CV events, beyond DR, DN, and CV risk factors (Table 2). There were no significant interactions between DR and DN in adjusted and unadjusted models. It has previously been reported that no significant interactions were detected with respect to prespecified patient subgroups for the ELIXA primary composite outcome, which included T2DM duration < 10 vs. >10 years [19]. In addition, no statistically significant interactions were found with respect to DR, DN, or duration of T2DM as a continuous variable.

### 3.3. Predictors of Retinopathy and/or Neuropathy

In both univariate and multivariate analysis, the most significant predictors of both DR and DN were duration of T2DM and insulin use (Table 3). The relationships between presence of DR and DN with duration of T2DM are shown in Supplementary Figures 2a–b. In multivariate analysis, other highly statistically significant (*p* < 0.001) predictors of DR were history of HF and stroke (Table 3) and of DN higher weight were previous percutaneous coronary intervention (PCI), total cholesterol, history of peripheral arterial disease, and previous stroke (Table 3). These models were effectively able to discriminate between patients with and without DR (area under the curve (AUC) 0.81) and DN (AUC 0.76).

## 4. Discussion

In this post hoc analysis of a large population selected for having a recent ACS event, together with T2DM, less than one-fourth of patients reported a history of DR, DN, or both. This was a smaller proportion than found in other studies of CV outcomes in T2DM, in which many patients had CV risk factors but not necessarily a completed event. For example, the prevalence of DR in the Trial to Reduce Cardiovascular Events with Aranesp Therapy (TREAT) study—which also relied on patient self-reports—was almost three times higher in a more severely compromised patient cohort with T2DM, chronic kidney disease, and anemia, but also twice as long T2DM duration [20]. The widely varying duration of T2DM in the ELIXA, and the inclusion of a large fraction with very short duration of T2DM, allowed our analysis to examine the relationships between duration of T2DM, presence of DR or DN, and risk of subsequent CV events.

The variability of previously reported associations between DR and/or DN and subsequent CV events could stem from variable T2DM duration and comorbidities of analyzed patient cohorts. The positive association of DR and CV events was observed in various patient cohorts [21–23]. In a recently published study with a large cohort of T2DM patients without the history of CV diseases, the risk of developing CV death, nonfatal MI, or stroke was about 40% higher in patients with DR and DN [24]. In the meta-analysis including 11,505 patients, DR was associated with 1.7-fold increased risk for CV events [25]. However, in the TREAT study, enrolling 4038 patients with T2DM, chronic kidney disease, and anemia, DR was not independently associated with a higher risk of renal or CV morbidity or death, possibly due to longer duration of T2DM and more comorbidities [20].

Our findings on DN are partially in accordance with previously published studies that revealed an association between autonomic DN and CV events [26]. Other studies have shown that DN was associated with a twofold risk increase for peripheral vascular disease [27]. In our study, we investigated the association of both DN and outcomes, but not peripheral vascular disease. Therefore, our findings on the large cohort of T2DM patients with recent ACS provide novel insight into the association between DN and recurrent CV events. These findings indicate the importance of the duration of T2DM as a traditional risk factor [28].

Consistent with prior reports, our analysis found that DR and/or DN were associated at baseline with longer T2DM duration, worse glycemic control, more insulin use, and history of prior manifest CV disease. Furthermore, both DR and DN were associated with increased risk of recurrent CV events, especially HF hospitalization. However, these associations were no longer statistically significant after adjustment for duration of T2DM, which remained a strong independent predictor of CV events [20, 29, 30]. Thus, DR and DN as well as duration of T2DM appear to be predictors of increased CV risk but, unlike diabetic nephropathy, not themselves are contributors to this risk.

While other relevant studies also adjusted clinical outcomes for the duration of T2DM—among other demographic characteristics and CV risk factors—none of them adjusted for only the known duration of the disease, which makes our findings unique. In a high-risk T2DM patient cohort used in the Veterans Affairs Diabetes Trial (VADT) (1791 subjects) [31], the duration of T2DM was significantly related to CV events. However, to the best of our knowledge, no studies included similar patient cohort, T2DM patients with recent ACS.

There are several possible explanations for the duration of T2DM being a strong independent predictor of recurrent CV events in our analysis. Longer duration of the disease may have a direct effect on progression of atherosclerotic lesions, increasing the risk of a recurrent CV event. In addition, it might be associated with autonomic DN and reduced heart rate variability, increasing the risk of CV death, which was not the case in our cohort. A long-term increase in oxidative stress in T2DM patients may cause increased risk of CV death. Also, longer exposure to hyperglycemia may simply reflect greater exposure to other, perhaps unmeasured, CV risk factors.

The major limitation of our study was the assessment of DR and DN which was based on patients' answers to self-report questionnaires. Therefore, the prevalence of both might have been underestimated. Such potential misclassification bias might have potentially weakened our results, and, therefore, the real association of DR and DN with recurrent CV events could be stronger than presented in this analysis. Another important limitation is the fact that T2DM duration was evaluated based on medical record or self-report and could therefore be underestimated, as patients often remain undiagnosed for many years. Also, having in mind that the study cohort included high-risk patients with T2DM and recent ACS, the results may not be applicable to other populations such as those without CV disease or with a less advanced stage of it. In addition, the design of this study was cross-sectional and therefore does not provide information on whether or not DR and DN developed before or after the onset of any CV disease antecedent to the qualifying ACS event. Finally, our findings should be considered post hoc and hypothesis-generating.

## 5. Conclusions

In a population with recent ACS together with T2DM, recurrent CV events, DR, and DN were all strongly associated with the T2DM duration. A history of either DR or DN was associated with increased risk of recurrent CV events. As these associations were eliminated by adjustment for the duration of T2DM, which remained a strong independent predictor of recurrent CV events, the link between DR and/or DN and these events is not likely to be a causal one. However, history of DR, DN, or both and longer duration of T2DM could be considered clinical markers for high risk of recurrent CV events. It is important to point out that the presence of both DN and DR is expected to positively correlate with patients' level of interaction with healthcare providers. Furthermore, it is expected that their level of healthcare provider interaction would correlate negatively with the frequency and magnitude of adverse outcomes.

## Figures and Tables

**Table 1:** Characteristics of all patients at baseline according to the presence of retinopathy and/or neuropathy.

Characteristic	All patients *n* = 6068	No retinopathy/neuropathy *n* = 4705	Retinopathy and/or neuropathy *n* = 1363	*p* value
Age (years)	60.3 ± 9.7	59.7 ± 9.7	62.3 ± 9.2	<0.001
Male sex (*n* (%))	4207 (69.3)	3379 (71.8)	828 (60.7)	<0.001
Body weight (kg)	84.9 ± 19.4	84.1 ± 18.9	87.6 ± 20.9	<0.001
Body mass index (kg/m^2^)	30.2 ± 5.7	29.9 ± 5.6	31.2 ± 6.0	<0.001
Duration of T2DM (years)	7.4 (2.8, 13.6)	6.0 (2.0, 11.7)	12.4 (7.0, 20.2)	<0.001
Categories of T2DM duration (*n* (%))				<0.001
≤1 year	964 (15.9)	914 (19.4)	50 (3.7)	
>1–≤5 years	1294 (21.3)	1130 (24.0)	164 (12.0)	
>5–≤10 years	1359 (22.4)	1076 (22.9)	283 (20.8)	
>10 years	2451 (40.4)	1585 (33.7)	866 (63.5)	
Race (*n* (%))				<0.001
White	4576 (75.4)	3471 (73.8)	1105 (81.1)	
Black	221 (3.6)	171 (3.6)	50 (3.7)	
Asian	771 (12.7)	669 (14.2)	102 (7.5)	
Other	500 (8.2)	394 (8.4)	106 (7.8)	
Region (*n* (%))				<0.001
Africa/Near East	296 (4.9)	228 (4.8)	68 (5.0)	
Asia Pacific	703 (11.6)	615 (13.1)	88 (6.5)	
Eastern Europe	1587 (26.2)	1115 (23.7)	472 (34.6)	
North America	807 (13.3)	564 (12.0)	243 (17.8)	
South and Central America	1944 (32.0)	1600 (34.0)	344 (25.2)	
Western Europe	731 (12.0)	583 (12.4)	148 (10.9)	
Smoking status (*n* (%))				<0.001
Current	709 (11.7)	579 (12.3)	130 (9.5)	
Former	2746 (45.3)	2184 (46.4)	562 (41.2)	
Never	2612 (43.1)	1941 (41.3)	671 (49.2)	
Diastolic blood pressure (mmHg)	77 ± 10	77 ± 10	76.3 ± 10	<0.001
Systolic blood pressure (mmHg)	130 ± 17	129 ± 17	131 ± 17	<0.001
Heart rate (beats/min)	70 ± 10	70 ± 10	71 ± 10	0.027
Fasting plasma glucose (mg/dl)	148.3 ± 51.6	145.2 ± 49.1	159.3 ± 58.1	<0.001
Glycated hemoglobin (%)	7.7 ± 1.3	7.6 ± 1.3	8.0 ± 1.2	<0.001
Glycated hemoglobin (mmol/mol)	61 ± 14	60 ± 14	64 ± 13	<0.001
Total cholesterol (mg/dl)	153.5 ± 44.6	151.3 ± 43.5	161.1 ± 47.3	<0.001
HDL cholesterol (mg/dl)	42.9 ± 10.9	42.6 ± 10.6	44.1 ± 11.7	<0.001
LDL cholesterol (mg/dl)	78.5 ± 35.3	77.1 ± 34.7	83.4 ± 36.9	<0.001
Triglycerides (mg/dl)	137.2 (99.1, 195.6)	136.3 (100.0, 192.9)	141.6 (99.1, 208.0)	0.021
eGFR (ml/min/1.73m^2^)	76 ± 21	77 ± 21	71 ± 22	<0.001
Albuminuria (*n* (%))				<0.001
<30 mg/g	4441 (74.3)	3579 (77.2)	862 (64.2)	
≥30–<300 mg/g	1148 (19.2)	819 (17.7)	329 (24.5)	
≥300 mg/g	389 (6.5)	237 (5.1)	152 (11.3)	
Medical history at randomization (*n* (%))				
Hypertension	4635 (76.4)	3463 (73.6)	1172 (86)	<0.001
Heart failure	1358 (22.4)	923 (19.6)	435 (31.9)	<0.001
Stroke	331 (5.5)	204 (4.3)	127 (9.3)	<0.001
Peripheral arterial disease	393 (6.5)	227 (4.8)	166 (12.2)	<0.001
Atrial fibrillation	366 (6.0)	247 (5.2)	119 (8.7)	<0.001
Percutaneous coronary intervention	4079 (67.2)	3263 (69.4)	816 (59.9)	<0.001
Coronary artery bypass grafting	507 (8.4)	363 (7.7)	144 (10.6)	<0.001
Qualifying ACS event (*n* (%))				<0.001
STEMI	2666 (44.0)	2187 (46.5)	479 (35.2)	
NSTEMI	2348 (38.7)	1817 (38.6)	531 (39.0)	
Unstable angina	1042 (17.2)	693 (14.7)	349 (25.6)	
Missing	9 (0.1)	6 (0.1)	3 (0.2)	
Antihyperglycemic therapy (*n* (%))				
Metformin	4243 (69.9)	3367 (71.6)	876 (64.3)	<0.001
Sulfonylureas	2266 (37.3)	1779 (37.8)	487 (35.7)	0.16
Insulin	2891 (47.6)	1948 (41.4)	943 (69.2)	<0.001
Thiazolidinediones	128 (2.1)	92 (2.0)	36 (2.6)	0.12
Alpha-glucose inhibitor	181 (3.0)	150 (3.2)	31 (2.3)	0.08
Dipeptidyl peptidase 4 inhibitor	226 (3.7)	176 (3.7)	50 (3.7)	0.90
Other	485 (8.0)	384 (8.2)	101 (7.4)	0.37

Data is presented as means ± SD, median (25–75th percentile), or percentages. HDL: high-density lipoprotein; LDL: low-density lipoprotein; MI: myocardial infarction; ACS: acute coronary syndrome; eGFR: estimated glomerular filtration rate.

**Table 2:** Multivariable modeling.

Hazard ratio (95% CI)		Primary composite endpoint *N* = 805 events	CV composite endpoint *N* = 913 events	Cardiovascular death *N* = 315 events	Myocardial infarction *N* = 531 events	Stroke *N* = 127 events	Heart failure hospitalization *N* = 249 events	Death *N* = 434 events
Univariate^∗∗^	Retinopathy	1.44 (1.19–1.75)^∗^	1.57 (1.31–1.88)^∗^	1.58 (1.17–2.13)^∗^	1.38 (1.08–1.76)^∗^	1.56 (0.97–2.51)	2.03 (1.48–2.78)^∗^	1.65 (1.28–2.12)^∗^
	Neuropathy	1.33 (1.12–1.57)^∗^	1.38 (1.19–1.62)^∗^	1.29 (0.99–1.68)	1.26 (1.02–1.54)^∗^	1.59 (1.07–2.37)^∗^	1.71 (1.30–2.27)^∗^	1.43 (1.15–1.78)^∗^
	T2DM duration (per 5 years)	1.17 (1.13–1.22) ^∗^	1.19 (1.15–1.23)^∗^	1.22 (1.16–1.29)^∗^	1.19 (1.14–1.25)^∗^	1.08 (0.98–1.19)	1.30 (1.23–1.38)^∗^	1.22 (1.17–1.28)^∗^

Model 1^∗∗^	Retinopathy	1.07 (0.86–1.32)	1.13 (0.94–1.31)	1.12 (0.80–1.56)	1.00 (0.77–1.31)	1.27 (0.74–2.16)	1.21 (0.85–1.73)	1.13 (0.85–1.50)
	Neuropathy	1.10 (0.92–1.31)	1.11 (0.94–1.31)	1.01 (0.76–1.34)	1.02 (0.82–1.28)	1.43 (0.93–2.20)	1.23 (0.90–1.66)	1.12 (0.88–1.42)
	T2DM duration (per 5 years)	1.16 (1.12–1.21)^∗^	1.18 (1.14–1.22)^∗^	1.21 (1.14–1.29)^∗^	1.19 (1.14–1.25)^∗^	1.04 (0.94–1.16)	1.28 (1.20–1.36)^∗^	1.21 (1.15–1.27)^∗^

Model 2^∗∗^	Retinopathy	1.07 (0.86–1.33)	1.13 (0.92–1.38)	1.12 (0.80–1.57)	1.00 (0.76–1.32)	1.28 (0.75–2.19)	1.15 (0.81–1.65)	1.15 (0.87–1.53)
	Neuropathy	1.02 (0.85–1.23)	0.99 (0.84–1.18)	0.91 (0.68–1.22)	1.00 (0.78–1.24)	1.27 (0.82–1.99)	0.96 (0.70–1.31)	1.03 (0.81–1.31)
	T2DM duration (per 5 years)	1.10 (1.05–1.15)^∗^	1.12 (1.07–1.16)^∗^	1.14 (1.07–1.22)^∗^	1.13 (1.08–1.20)^∗^	0.95 (0.85–1.07)	1.21 (1.13–1.30)^∗^	1.13 (1.07–1.19)^∗^

Primary composite endpoint: CV death, nonfatal MI, stroke, or hospitalization for unstable angina; CV composite endpoint: CV death, nonfatal MI, stroke, hospitalization for heart failure (HF); ^∗^ *p* ≤ 0.05. Univariate: adjusted for randomized study treatment only; model 1: adjusted for retinopathy, neuropathy, T2DM duration, and randomized study treatment; model 2: model 1+ age, sex, race, BMI, baseline HbA1c, smoking, history of hypertension, heart rate, total cholesterol, LDL cholesterol, and triglycerides; ^∗∗^ *p* ≤ 0.05.

**Table 3:** Predictors of retinopathy and neuropathy multivariate models.

Parameter	Retinopathy		Neuropathy	
	OR (95% CI)	∣*Z*∣ score	OR (95% CI)	∣*Z*∣ score
Duration of diabetes (per 5 years)	1.48 (1.41–1.56)	14.64	1.31 (1.25–1.37)	11.52
Insulin use	2.77 (2.23–3.46)	9.13	2.19 (1.86–2.58)	9.36
Weight (per 5 kg)	—	—	1.09 (1.07–1.11)	8.83
Previous PCI	0.75 (0.61–0.91)	2.94	0.60 (0.51–0.70)	6.32
Total cholesterol (per 10 mg/dl)	—	—	1.05 (1.04–1.07)	6.23
History of PAD	—	—	1.88 (1.45–2.43)	4.82
History of HF	1.65 (1.35–2.02)	4.82	1.32 (1.12–1.56)	3.23
Previous stroke	2.06 (1.51–2.82)	4.54	1.86 (1.42–2.45)	4.45
Diastolic blood pressure (per 10 mmHg)	—	—	0.88 (0.82–0.95)	3.25
Sulphonylurea use	—	—	1.29 (1.11–1.51)	3.24
Previous CABG	0.72 (0.52–0.98)	2.11	0.66 (0.51–0.86)	3.06
Presence of hypertension	1.46 (1.11–1.91)	2.69	1.39 (1.12–1.72)	3.00
Body mass index (per 5 kg/m^2^)	1.12 (1.04–1.22)	2.99	—	—
Glucose (per 10 mg/dl)	1.02 (1.0-1.04)	2.29	1.02 (1.01–1.03)	2.87
eGFR (per 10 ml/min/1.73m^2^)	0.94 (0.90–0.98)	2.66	—	—
Male sex	—	—	1.25 (1.06–1.47)	2.60
HDL cholesterol (per 10 mg/dl)	1.11 (1.02–1.20)	2.53	—	—
Age (per 5 years)	—	—	1.05 (1.01–1.10)	2.20
HbA1c (per 1%)	1.09 (1.00–1.19)	1.98	—	—

Model AUC = 0.81 (retinopathy) and model AUC = 0.76 (neuropathy). HF: heart failure; PCI: percutaneous coronary intervention; eGFR: estimated glomerular filtration rate; HDL: high-density lipoprotein; CABG: coronary artery bypass grafting; HbA1c: glycated hemoglobin; PAD: peripheral artery disease.

## Data Availability

The data used to support the findings of this study are available from the corresponding author upon reasonable request.

## Supplementary Materials

Supplementary Materials Supplementary Figure 1. Patient distribution by T2DM duration categories widely varies: 15.9 % of participants have known T2DM for less than 1 year, 21.3% ≤5 years, 22.4% >1–≤5 years, and 40.4% longer than 10 years. Supplementary Figures 2a–b. Relationship of retinopathy and neuropathy with T2DM duration.
