# Supplementary material for: Retinopathy, Neuropathy, and Subsequent Cardiovascular Events in Patients with Type 2 Diabetes and Acute Coronary Syndrome in the ELIXA: The Importance of Disease Duration
Source: J Diabetes Res. 2018 Dec 16;2018:1631263. doi: 10.1155/2018/1631263 (PMC6311850; doi:10.1155/2018/1631263)
Supplement: Supplementary Materials — Supplementary Figure 1. Patient distribution by T2DM duration categories widely varies: 15.9 % of participants have known T2DM for less than 1 year, 21.3% ≤5 years, 22.4% >1–≤5 years, and 40.4% longer than 10 years. Supplementary Figures 2a–b. Relationship of retinopathy and neuropathy with T2DM duration. [file 1631263.f1.docx]

**SUPPLEMENTARY MATERIAL**

**Figure Legends**

**Supplementary Figure 1.** Patient distribution by T2DM duration categories widely varies: 15.9 % of participants have known T2DM for less than 1 year, 21.3% ≤5 years, 22.4% >1-≤5 years, and 40.4% longer than 10 years.

**Supplementary Figure 2a-b**. Relationship of retinopathy and neuropathy with T2DM duration

**
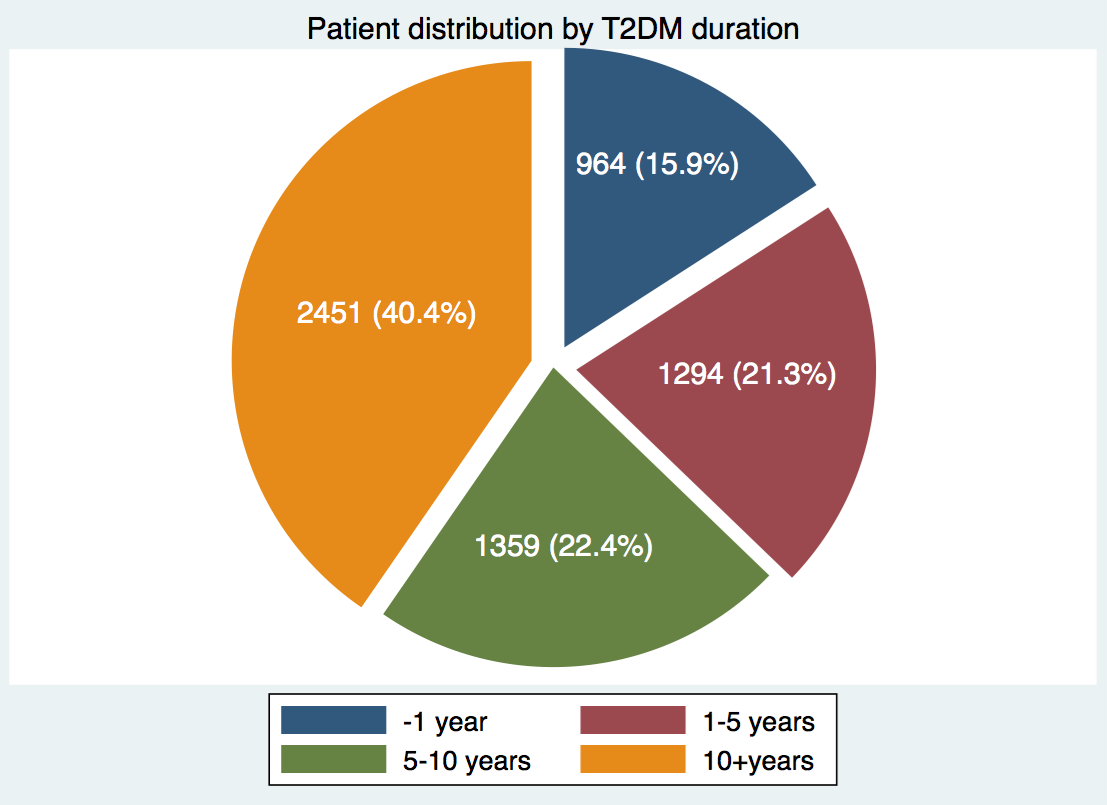
**
